# Epidemiology, validation, and clinical characteristics of inflammatory bowel disease: the ABIS birth cohort study

**DOI:** 10.1186/s12876-023-02840-1

**Published:** 2023-06-08

**Authors:** Malin Östensson, Olle Björkqvist, Annie Guo, Ketil Størdal, Jonas Halfvarson, Karl Mårild, Johnny Ludvigsson

**Affiliations:** 1 Bioinformatics and Data Centre, Sahlgrenska Academy, University of Gothenburg, Gothenburg, Sweden; 2 Department of Laboratory Medicine, Clinical Microbiology, Faculty of Medicine and Health, Örebro University, Örebro, Sweden; 3 Department of Paediatrics, Institute of Clinical Science, University of Gothenburg, Gothenburg, Sweden; 4 Department of Paediatric Research, Faculty of Medicine, University of Oslo, Oslo, Norway; 5 Children’s Centre, Oslo University Hospital, Oslo, Norway; 6 Department of Gastroenterology, Faculty of Medicine and Health, Örebro University, Örebro, Sweden; 7 Department of Paediatrics, Queen Silvia Children’s Hospital, 416 78 Gothenburg, Sweden; 8 Crown Princess Victoria Children’s Hospital, Region Östergötland, Linköping, Sweden; 9 Department of Biomedical and Clinical Sciences, Division of Paediatrics, Linköping University, Linköping, Sweden

**Keywords:** Inflammatory bowel disease, All Babies in Southeast Sweden, Incidence, Validation, Swedish National Patient Register

## Abstract

**Background:**

Birth cohort studies with linked register-based data on inflammatory bowel disease (IBD) provide opportunities to prospectively study early-life determinants of the disease. However, register-based data often lack information on clinical characteristics and rely on diagnostic algorithms. Within the All Babies in Southeast Sweden (ABIS) cohort, we examined the validity of a register-based definition of IBD, its incidence, and clinical and therapeutic characteristics at diagnosis.

**Methods:**

We followed 16,223 children from birth (1997–1999) until the end of 2020 for the diagnosis of IBD as defined by a minimum of two diagnostic codes for IBD in the Swedish National Patient Register (NPR). We described the incidence and cumulative incidence of IBD. Through a medical record review of cases diagnosed by the end of 2017, we examined the positive predictive value (PPV) for IBD and described its clinical characteristics and treatment.

**Results:**

By 2020, at an average age of 22.2 years, 113 participants (0.74%, 95% confidence interval [CI] = 0.61–0.89) had a register-based diagnosis of IBD, corresponding to an incidence of 31.3 per 100,000 person-years of follow-up; the incidence for Crohn’s disease (CD) was 11.1 per 100,000 person-years and 15.8 for ulcerative colitis (UC). Of 77 participants with a register-based definition of IBD by the end of 2017, medical records were identified for 61 participants, of whom 57 had true IBD (PPV = 93%; 95%CI = 0.87–1.00). While oral 5-aminosalicylic acid treatment was equally common in newly diagnosed CD and UC patients, biologics were more often used for newly diagnosed CD. The median faecal calprotectin levels were 1206 mg/kg at diagnosis and 93 mg/kg at the last follow-up (*P* < 0.001).

**Conclusions:**

In this population-based sample of Swedish children and young adults the cumulative IBD incidence was 0.74. The validity of register-based definition of IBD was high and supports using such data to identify IBD patients in cohort studies.

**Supplementary Information:**

The online version contains supplementary material available at 10.1186/s12876-023-02840-1.

## Background

Inflammatory bowel disease (IBD), mainly consisting of Crohn's disease (CD), ulcerative colitis (UC) [1], is a life-long and life-limiting disease characterised by inflammation of the gastrointestinal tract, particularly in the small intestine and colon [2]. About 25% of all IBD diagnoses present before adulthood [3]. Compared to adults, childhood-onset IBD has been linked to a more severe disease course, e.g., cancer [4], growth failure [5, 6], and a negative impact on school performance and health-related quality of life [7, 8].

Over the past decades, there has been a considerable rise in the incidence of IBD worldwide, with the highest risks reported from Nordic countries (paediatric estimates summarised in Additional file 1: Table 1). Although there is ample evidence that environmental exposures contribute to IBD [9], the nature and timing of such factors remain unclear. Prospective population-based birth cohorts provide opportunities to study early-life environmental candidates for IBD (e.g., diet and hygiene-related factors) [10]. While linkages to register-based IBD data may reduce the impact of attrition within longitudinal cohorts, register-based IBD data usually lack detailed information on clinical features and rely on the validity of diagnostic algorithms. Because disease heterogeneity has been one of the hallmarks of IBD, precise information on clinical disease characteristics offers the possibility of studying risk factors for distinctive IBD phenotypes [11].

Two Swedish studies have found the validity of a commonly used register-based IBD definition to be high (positive predictive values [PPVs] 93–95%) [12, 13]. However, these validation studies have only included adult-onset IBD or were confined to childhood-onset IBD diagnosed in tertiary care university hospitals. The performance of IBD algorithms may differ between healthcare settings and between children and adults [12].

Using data from a population-based birth cohort, we sought to examine the validity of a register-based definition of IBD, its incidence, and clinical and therapeutic properties at diagnosis.

## Methods

Based on the All Babies in Southeast Sweden (ABIS) birth cohort, this study used questionnaire data collected at birth, linked data on IBD diagnoses recorded in the Swedish National Patient Register (NPR) [14], and clinical data retrieved from medical records.

### Study population

**Fig. 1:**
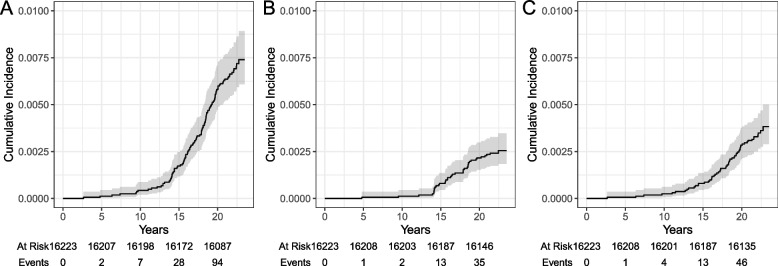
Figure illustrating the age-specific cumulative incidence of a register-based diagnosis of inflammatory bowel disease (**A**), Crohn's disease (**B**), and ulcerative colitis (**C**). Gray area indicates 95% confidence intervals

ABIS is a prospective population-based cohort study in which all 21,700 women who gave birth in Southeast Sweden between October 1, 1997 and October 1, 1999 were asked to participate. The cohort includes data on 17,055 children (78.6% participation rate) [15]. For this study, we restricted participation to 16,223 children with any questionnaire data and a valid personal identification number enabling register linkages [16] (A flowchart is presented in Additional file 1; Fig. 1).

We included parent-reported at-birth questionnaire data on the child's sex, birth year, gestational age, small for gestational age (< 2 standard deviations of mean weight for age), parental IBD, maternal education level, and delivery mode. Information on maternal age at delivery was collected from the Medical Birth Register [17]. Data were categorised as shown in Table 1.

**Table 1:** Characteristics of participants with a register-based diagnosis of inflammatory bowel disease in the ABIS cohort

**Characteristic**	**Overall, *N* = 16,223**	**No IBD, *n* = 16,110**	**IBD**^**a**^**, *n* = 113**	**CD, *n* = 40**	**UC, *n* = 57**
**Sex**					
Female	7,821 (48.2%)	7,769 (48.2%)	52 (46.0%)	14 (35.0%)	27 (47.4%)
Male	8,402 (51.8%)	8,341 (51.8%)	61 (54.0%)	26 (65.0%)	30 (52.6%)
**Birth year**					
1997	1,668 (10.3%)	1,657 (10.3%)	11 (9.7%)	3 (7.5%)	6 (10.5%)
1998	8,602 (53.0%)	8,536 (53.0%)	66 (58.4%)	25 (62.5%)	33 (57.9%)
1999	5,953 (36.7%)	5,917 (36.7%)	36 (31.9%)	12 (30.0%)	18 (31.6%)
**Age at end of observation/diagnosis (years)**					
Mean (SD)	22.2 (1.0)	22.2 (0.9)	16.9 (3.7)	16.5 (3.3)	17.0 (4.1)
Median (IQR)	22.3 (21.7, 22.7)	22.3 (21.8, 22.7)	17.9 (15.1, 19.5)	16.4 (14.3, 18.5)	18.1 (15.7, 19.7)
Range	0.1, 23.5	0.1, 23.5	2.6, 22.7	4.7, 22.4	2.6, 22.7
**Maternal IBD**					
Yes	99 (0.6%)	95 (0.6%)	4 (3.5%)	2 (5.0%)	2 (3.5%)
**Paternal IBD**					
Yes	98 (0.6%)	97 (0.6%)	1 (0.9%)	1 (2.5%)	0 (0.0%)
**Maternal education level**					
0–9 years	1,358 (8.6%)	1,346 (8.6%)	12 (10.7%)	5 (12.5%)	7 (12.5%)
10–12 years	8,813 (55.6%)	8,749 (55.6%)	64 (57.1%)	20 (50.0%)	33 (58.9%)
≥ 13 years	5,669 (35.8%)	5,633 (35.8%)	36 (32.1%)	15 (37.5%)	16 (28.6%)
*Missing data*	383	382	1	0	1
**Delivery mode**					
Vaginal	13,040 (80.4%)	12,949 (80.4%)	91 (80.5%)	34 (85.0%)	45 (78.9%)
Caesarean	1,866 (11.5%)	1,851 (11.5%)	15 (13.3%)	4 (10.0%)	8 (14.0%)
*Missing data*	1317 (8.1%)	1310 (8.1%)	7 (6.2%)	2 (5.0%)	4 (7.0%)
**Maternal age at delivery (years)**					
Mean (SD)	29.0 (4.6)	29.0 (4.6)	29.3 (4.6)	29.4 (5.2)	28.8 (4.2)
Missing data	250	250	0	0	0
**Gestational age (weeks)**					
Mean (SD)	39.7 (1.8)	39.7 (1.8)	39.6 (1.7)	39.7 (1.8)	39.7 (1.6)
*Missing data*	333	331	2	0	1
**Small for gestational age**^**b**^					
Small	292 (1.9%)	288 (1.9%)	4 (3.6%)	2 (5.1%)	1 (1.8%)
*Missing data*	732	729	3	1	1

^a^Include data on patients with undefined inflammatory bowel disease (IBD), where a mix of IBD codes did not allow us to distinguish Crohn's disease (CD) from ulcerative colitis (UC)

^b^Small for gestational age equalled < 2 standard deviations (SD) of mean weight for age. *ABIS* All Babies in Southeast Sweden, *IQR* interquartile range

### Register-based definition of IBD

Similar to others [18–21], our register-based definition of IBD required a minimum of two diagnostic listings of IBD recorded in the NPR until December 31, 2020 (International Classification of Diseases [ICD] codes are detailed in Additional file 1: Table 2). In line with previous works [22] we used subtype-specific ICD-10 codes to categorise patients into CD and UC based on their most recent diagnosis over the past 5 years of follow-up. Patients with undefined IBD codes over follow-up were defined as undefined IBD. The Swedish NPR started in 1964, became nationwide in 1987, and since 2001 includes diagnostic and procedure codes for all inpatient and hospital-based outpatient care in Sweden. The NPR has been shown to contain high-quality data, as described elsewhere [23].

### Validation of IBD and characterisation of disease phenotype

To evaluate the predictive performance of our register-based IBD definition we requested copies of the medical records for all 77 patients with ≥ 2 ICD codes for IBD by December 2017, i.e., confined mostly to childhood-onset IBD (< 18 years). Medical records were primarily collected from the departments of paediatric and the departments of medicine in the region(s) from which the ICD codes had been registered.

From the medical records, the first author (OB; Ph.D. Student and physician) collected data on symptoms, endoscopy findings, histology, and radiology reports to determine whether the participant had true IBD using internationally accepted criteria for IBD as the gold standard [24]. Patients whose diagnostic data were incompletely recorded were still regarded as having true IBD if notes written by the treating physician indicated that the patient fulfilled the diagnostic criteria for IBD. For discrepant diagnostic data, validation assessments were resolved in discussion with a second author (JH; IBD specialist clinician).

The Montreal classification was used to define disease phenotype for validated (true) IBD cases [25]. In addition, information on C-reactive protein (CRP), faecal calprotectin, IBD-related medications, surgery, and extraintestinal manifestations were collected from patient medical records. Clinical and therapeutic data were retrieved from the first year of diagnosis; laboratory data were recovered from the last recorded measurement in the medical records.

### Statistical analyses

Incidence rates of overall IBD and the subtypes (UC and CD) were estimated based on follow-up from birth until IBD diagnosis, defined as the time of the first-ever listing of IBD or by the end of data capture on December 31, 2020. Kaplan–Meier curves illustrated the age-specific incidence of IBD, CD, and UC. The cumulative incidence of IBD, CD, and UC was calculated based on the number of ABIS participants with a register-based definition of IBD by December 31, 2020. The Wilcoxon rank sum test was used for comparisons of calprotectin and CRP levels. The statistical analyses were performed using R statistical software (v4.2.1) [26].

## Results

We followed 16,223 participants (7821 women [48.2%]) throughout 2020 (average age 22.2 years, SD 1.0; Table 1).

**Fig. 2:**
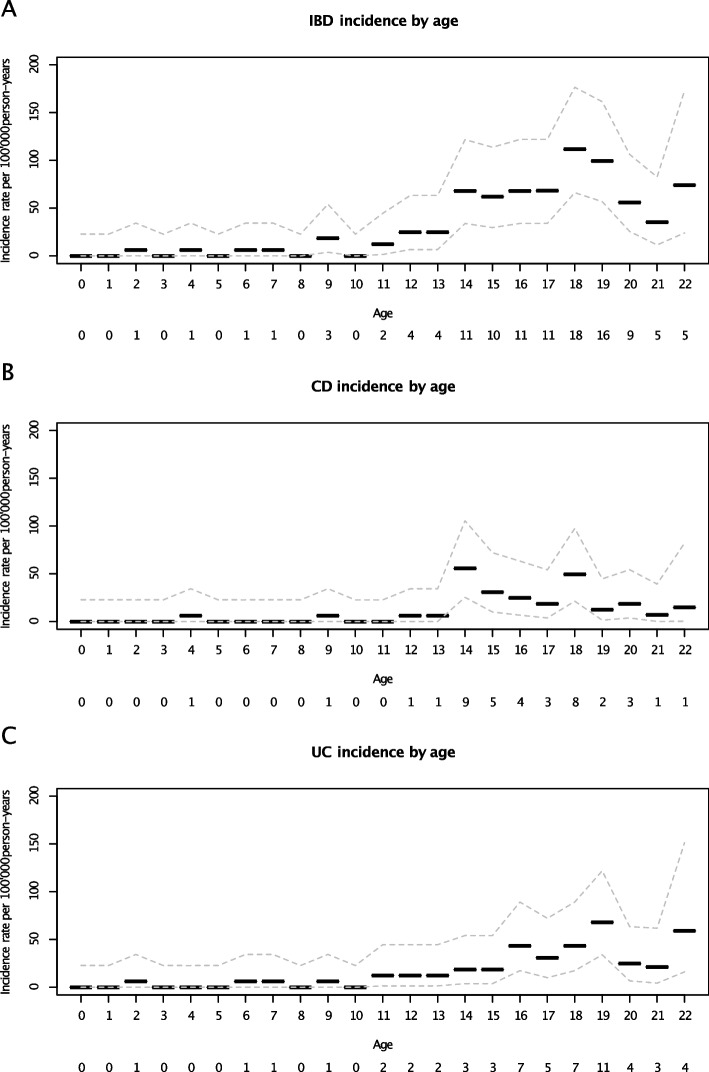
Age-specific incidence of register-based diagnosis of inflammatory bowel disease (IBD), Crohn's disease (CD), and ulcerative colitis (UC). Events per age are reported below the x-axis. Our IBD definition required at least of two diagnostic codes for IBD. Hence, the incidence may have been underestimated during the end of the study period

By the end of 2020, over 360,655 person-years (PYR) of follow-up, 113 participants had a register-based IBD diagnosis corresponding to an incidence rate of 31.3 per 100,000 PYR (Table 2). The incidence rate for CD was 11.1 per 100,000 PYR and for UC 15.8. The incidence of IBD increased markedly from 10 years of age, with a peak incidence at age 15 years (Figs. 1 and 2). Age-specific incidence rates for CD and UC were also highest during adolescence. While parental IBD was more common in participants with IBD than those without IBD, perinatal characteristics were broadly similar between study participants (Table 1).

**Table 2:** Incidence rate of register-based diagnosis of inflammatory bowel disease, Crohn's disease, and ulcerative colitis

**Disease**	**Person-years of follow-up**	**Events**	**Incidence (95%CI)**	**Cumulative incidence (95%CI)**
**IBD**	360,655	113	31.3 (25.8, 37.7)	0.74 (0.61, 0.89)
**CD**	361,537	40	11.1 (7.9, 15.1)	0.26 (0.19, 0.35)
**UC**	361,468	57	15.8 (11.9, 20.4)	0.38 (0.29, 0.50)

Data based on the All Babies In Southeast Sweden (ABIS) cohort until December 31, 2020

*CI* confidence interval, *CD* Crohn's disease, *IBD* inflammatory bowel disease, *UC* ulcerative colitis

On December 31, 2020, 113 participants had IBD according to the register-based definition (i.e. ≥ 2 diagnostic listings of IBD in the NPR); of these 113 participants, 40 were defined as CD and 57 as UC, corresponding to a cumulative incidence of 0.74% (95% confidence interval [CI] 0.61–0.89) for IBD, 0.26% (95% CI 0.19–0.35) for CD, and 0.38% (95% CI 0.29–0.50) for UC (Table 2).

### Validity of register-based definition of IBD

To investigate the validity of the register-based IBD definition we requested copies of the medical records for the 77 participants with a minimum of two listings of IBD by December 31, 2017. Medical records were successfully retrieved for 61 participants; for 16 patients with at least two diagnostic listings of IBD, we could not recover copies of the medical records because of local regulations or failure to identify the clinic that had registered the ICD codes.

Of 61 participants with medical records, 57 had a true (confirmed) IBD, resulting in a PPV of 93% (95% CI 0.87–1.00) for a diagnosis of IBD. The four participants whose medical records were reviewed and who did not have IBD had infectious gastroenteritis or unspecific intestinal inflammation.

To assess the potential impact of ascertainment bias of medical records we also estimated PPV for IBD, assuming that all 16 participants with missing medical records did not have IBD. Under this assumption, the PPV for IBD was 74% (95% CI 0.64–0.84).

### Clinical characteristics from medical record review

For the 57 patients whose IBD diagnosis was confirmed by medical record review an overview of the clinical characteristics at diagnosis is presented in Additional file 1, Tables 3–5. In CD patients disease location was evenly distributed between the terminal ileum (33%, *n* = 9/27), colon (37%, *n* = 10/27), and ileocolonic involvement (36%, *n* = 7/27). Eleven per cent (*n *= 3/27) of CD patients had complicated disease behaviour (B2 stricturing or B3 penetrating). Most of the patients with UC had extensive colitis (52%, *n* = 14/27), followed by proctitis (22%, *n* = 6/27) and left-sided colitis (19%, *n* = 5/19).

**Table 3:** Medical therapy^a^ for inflammatory bowel disease within the first year of diagnosis

**Therapy**	**Crohn's disease** ***N* = 27**	**Ulcerative colitis** ***N* = 27**
**Oral corticosteroids**		
Yes	24 (89%)	15 (56%)
No	1 (4%)	8 (30%)
*Missing data*	2 (7%)	4 (15%)
**Oral 5-ASA**		
Yes	18 (67%)	17 (63%)
No	7 (26%)	6 (22%)
*Missing data*	2 (7%)	4 (15%)
**Azathioprine**		
Yes	21 (78%)	9 (33%)
No	4 (15%)	14 (52%)
*Missing data*	2 (7%)	4 (15%)
**Biologics**		
Yes	9 (33%)	1 (4%)
No	16 (59%)	22 (81%)
*Missing data*	2 (7%)	4 (15%)

^a^Data restricted to systemic medical therapy of 57 patients who had a validated (true) inflammatory bowel disease diagnosis on medical record review. Three patients with undefined inflammatory bowel disease (IBD) were not specifically reported in the table: three had oral corticosteroids, two with undefined IBD had 5-aminosalicylic acid (5-ASA), one had azathioprine and none of those three with undefined IBD had biologics

During the first year of diagnosis, oral 5-aminosalicylic acid use was essentially equal among patients with CD (*n* = 18, 67%) and UC (*n* = 17, 63%; Table 3). Treatment characteristics stratified by age are presented in Additional file 1, Table 6–7. In contrast, oral corticosteroids, azathioprine, and biologics were more common in patients with CD. Only one patient underwent ileocecal resection during the first year of CD diagnosis. In IBD patients with information on faecal calprotectin at diagnosis and last follow-up (*n* = 38), the median faecal calprotectin levels were 1206 mg/kg at diagnosis and 93 mg/kg at the last follow-up (*P* < 0.001; median time between first and last record, 3.7 years; IQR 1.9–5.0 years). The median CRP levels were 10 mg/L at diagnosis and 5 mg/L at the last follow-up (*P* < 0.001, *n* = 25; median time between the first and last record, 4.2 years, IQR: 3.0–5.3 years).

## Discussion

In the population-based ABIS cohort, followed from birth to a mean age of 22 years, the incidence of IBD was 31.3 per 100,000 PYR. By December 2020, the cumulative IBD incidence was 0.74%. We found that at least two IBD diagnostic listings in the NPR were very consistent with a clinical diagnosis of IBD (PPV 93%), enabling register-based IBD diagnosis for use in the ABIS study and other similar cohorts.

The Nordic countries have one of the highest incidence rates of childhood-onset IBD [27]. However, estimates have varied widely, ranging from 4.3 to 23.0 per 100,000 PYR (previous IBD estimates from Nordic countries are summarised in Additional file 1: Table 1). These reported discrepancies in IBD risk may be attributed to population characteristics (e.g., age) and variations in definitions of IBD and how IBD was identified (register-based vs medical records) [28–33]. The sharp increase in incidence in late teenage years must be considered when comparing studies with different age cut-offs. Improved diagnostic strategies, such as increased use of faecal calprotectin as a non-invasive marker for IBD, could also contribute to a slightly earlier diagnosis.

In this cohort the incidence of IBD was 31.3 per 100,000 PYR of follow-up, with a peak incidence in late adolescents. The cumulative incidence of IBD was 0.74%. The observed risk of IBD in the ABIS cohort was markedly higher compared to Nordic studies restricted to childhood-onset IBD (Additional file 1: Table 1) [18]. A Danish Crohn's Colitis database study reported incidence rates of CD and UC to be over three times as high in 16–25-year-olds compared to those < 15 years [32]. Differences in incidence rates can also be affected by disparities in time trends of IBD between countries. For instance, although Sweden and Denmark share remarkable similarities in lifestyles and living conditions, Sweden developed an increasing incidence rate of CD and UC almost 15 years before Denmark [34, 35].

The incidence of UC was higher than CD in our cohort of children and young adults. While a similar UC predominance has been reported from Northern Europe [36], most other paediatric studies [27], including observations from Denmark [37], Norway [38], and Scotland [39], have shown a higher incidence of CD compared to UC. Still, the comparison of UC:CD ratios across studies is challenging. For instance, differences in IBD subtype predominance between studies are partly related to the length of follow-up, as IBD subtypes can be difficult to determine, especially at diagnosis. In a Swedish register-based study almost one in five patients changed IBD subtype during follow-up [40], and in this and other studies the proportion of CD increased and UC decreased when defined at the start or end of the follow-up [41, 42]. Thus, the UC:CD ratio in the ABIS cohort may change with longer follow-ups since the diagnosis of IBD. On the other hand, the relative incidence of CD and UC varies across age groups [27]. A shift from a predominance of CD to UC is expected with increasing age of the study population. Most Nordic studies of adult patients have reported a higher incidence of UC than CD [43].

The reason for the risk of IBD worldwide, and particularly in Nordic countries, remains to be explained. Although genetics are important for IBD development [44], genetic predisposition cannot alone fully explain the trend for the increased incidence of IBD. Instead, the increase in IBD incidence over the past decades parallels our changing lifestyle and underscores the role of the environment in disease development [45]. The added emphasis stems from twin and migration studies highlighting early-life events as critical for IBD development [9, 46]. Identification of these important yet poorly defined environmental factors is key to the future prevention of IBD. Research on the environmental determinants of IBD has been hampered by a scarcity of prospective data from sufficiently powered, population-based cohorts. However, the few prospective studies available are limited to adults [47]. Linking high-quality register-based outcome data to cohorts allows the study of relatively rare events while minimising bias due to loss of follow-up. Register data may be especially important for IBD, given the notion of a considerable latency period between exposure and IBD onset [48].

When validating a commonly used register-based definition of IBD, 57 of 61 participants in this study were confirmed to have IBD on medical records, corresponding to a PPV of 93%. In 2017, Jakobsson et al*.* validated adult IBD diagnoses in the Swedish Quality Register for IBD [49], finding a PPV of 93% for IBD but somewhat lower PPVs for IBD subtypes. More recently, Mouratidou et al*.* reported a PPV of 93% for childhood-onset IBD with at least two diagnostic IBD listings in the Swedish NPR [12] and an even higher PPV for patients with pathology reports consistent with IBD [50].

While our findings align with these previous Swedish validation studies [12, 49], our data go beyond by showing a similar validity of register-based IBD in a cohort of Swedish children and young adults often diagnosed outside tertiary care settings. Moreover, Danish NPR data have shown a high PPV for IBD in the medical records of adults [50], with increasing PPV with a higher number of IBD listings, ranging from a PPV of 78% for one IBD listing to a PPV of 90% for at least two listed IBD diagnoses. Also, Swedish data have shown a PPV for IBD of only 74% with one diagnostic listing of IBD, which implies that a register-based IBD algorithm using one IBD listing would yield a high proportion of false positives, hampering cohort studies on IBD. The high quality of IBD diagnostic data of the NPR shown in this and other Swedish studies agrees with validation data on many, but not all, chronic diseases with PPV ranging from 85 to 95% [23].

### Strengths and limitations

This study has several strengths, including using register-based and electronic medical record data to describe the epidemiology and clinical phenotype of IBD in children and young adults. Using a sample from the population-based, large-scale ABIS cohort minimises the risk of selection bias and ensures high generalisation of our results across similar populations and healthcare settings. In addition, diagnoses from the ABIS cohort were confirmed through an extensive review of medical records obtained from regional and tertiary care university hospitals.

This study has several limitations. First, we could not recover the medical records for 16/77 (21%) requested participants with a register-based IBD diagnosis. Consequently, our verification was based on a subgroup of IBD patients identified in ABIS, and it is unknown how missing data may have affected our results. Second, we could not check the PPVs for specific IBD subtypes because of limited sample size. Finally, the validation of IBD diagnoses was not blinded and was essentially limited to childhood-onset cases diagnosed by the end of 2017.

## Conclusions

In this cohort of children and young adults the incidence of IBD was 31.3 per 100,000 PYR of follow-up, with a cumulative IBD incidence of 0.74%. We found a minimum of two IBD diagnostic listings in the NPR to be highly consistent with a clinical IBD diagnosis which supports using NPR data to identify IBD patients in cohort studies.

## Supplementary Information

**Additional file 1.**

## Data Availability

Data are available from the corresponding author upon reasonable request.

## Abbreviations

ABIS: All Babies in Southeast Sweden
CRP: C-reactive protein
CI: Confidence interval
CD: Crohn's disease
IBD: Inflammatory bowel disease
IQR: Interquartile range
PYR: Person-year
PPV: Positive predictive value
SD: Standard deviation
UC: Ulcerative colitis
