# Supplementary material for: Epidemiology, validation, and clinical characteristics of inflammatory bowel disease: the ABIS birth cohort study
Source: BMC Gastroenterol. 2023 Jun 8;23:199. doi: 10.1186/s12876-023-02840-1 (PMC10249249; doi:10.1186/s12876-023-02840-1)
Supplement: Supplementary file 1 — Additional file 1. [file 12876_2023_2840_MOESM1_ESM.docx]

*Additional file 1 to*

**Epidemiology, Validation, and Clinical Characteristics of Inflammatory Bowel Disease: The ABIS birth cohort study**

*By Malin Östensson and Olle Björkqvist et al.*

**Article search**

A PubMed search was performed on June 14, 2022 using the search string presented below. Inclusion criteria were full-text, peer-reviewed English articles reporting original data on the incidence and/or prevalence of inflammatory bowel disease, ulcerative colitis, and Crohn's disease in children/young adults (aged 0-29 years) living in Nordic countries. We restricted our literature search to studies published since the year 2000. Reference lists and citations of relevant papers found in the literature search were screened to broaden the number of relevant articles.

(("paediatrics"[All Fields] OR "pediatrics"[MeSH Terms] OR "pediatrics"[All Fields] OR "paediatric"[All Fields] OR "pediatric"[All Fields]) AND ("inflammatory bowel diseases"[MeSH Terms] OR ("inflammatory"[All Fields] AND "bowel"[All Fields] AND "diseases"[All Fields]) OR "inflammatory bowel diseases"[All Fields] OR ("inflammatory"[All Fields] AND "bowel"[All Fields] AND "disease"[All Fields]) OR "inflammatory bowel disease"[All Fields]) AND ("epidemiology"[MeSH Subheading] OR "epidemiology"[All Fields] OR "incidence"[All Fields] OR "incidence"[MeSH Terms] OR "incidences"[All Fields] OR "incident"[All Fields] OR "incidents"[All Fields]) AND ("epidemiology"[MeSH Subheading] OR "epidemiology"[All Fields] OR "prevalence"[All Fields] OR "prevalence"[MeSH Terms] OR "prevalance"[All Fields] OR "prevalences"[All Fields] OR "prevalence s"[All Fields] OR "prevalent"[All Fields] OR "prevalently"[All Fields] OR "prevalent"[All Fields])) AND (clinicaltrial[Filter] OR meta-analysis[Filter] OR randomizedcontrolledtrial[Filter])

The literature search yielded 78 articles that were screened by second author AG for eligibility. Reference lists and citations of included papers were then screened to identify additional relevant articles. A total of 23 articles were found relevant for the study and summarised in Table 1.

**Table 1.** Nordic incidence and prevalence studies of paediatric inflammatory bowel disease published since 2000 (n=23)

| **Country (region)** | **Study**  **author** | **Calendar years of IBD diagnosis** | **Source of diagnosis** | **Event, n** | **Age (years)** | **Incidence per**  **100,000 PYR** | | | **Prevalence per**  **100,000** | | |
| --- | --- | --- | --- | --- | --- | --- | --- | --- | --- | --- | --- |
|  |  |  |  |  |  | **IBD** | **CD** | **UC** | **IBD** | **CD** | **UC** |
| Denmark (North Jutland County) | Jakobsen et al [1] (2006) | 1978 – 2002 | Health records | 95 | <15 | - | 1.3 (females) 1.6 (males) | 3.2 (females) 2.1 (males) | - | - | - |
|  |  |  |  | 762 | 15 – 29 | - | 19.2 (females) 15.6 (males) | 15.4 (females) 8.8 (males) | - | - | - |
| Denmark (Eastern County) | Jakobsen et al [2] (2008) | 1998 – 2000 | Health records | 98 | <15 | 4.3 | 2.3 | 1.8 | 15.8 | 6.7 | 8.3 |
|  |  | 2002 – 2004 |  | 145 |  | 6.1 | 3.1 | 2.7 | 20.3 | 8.2 | 10.5 |
| Denmark (Copenhagen) | Jakobsen et al [3] (2009) | 1998 – 2006 | Health records | 50 | <15 | 4.7 | 3.1 | 1.6 | - | - | - |
| Denmark | Larsen et al [4] (2016) | 1995 – 2013 | NPR | 2424 | <17 | - | 10.0 (females)  9.4  (males) | 7.2 (females) 6.2  (males) | - | - | - |
| Denmark (North Denmark) | Larsen et al. [5] (2023) | 1978 - 2020 | NPR | 6158 | <15 | 7.6 | - | - | - | - | - |
|  |  |  |  |  | 15-29 | 56.3 | - | - | - | - | - |
| Denmark | Lophaven et al [6] (2017)^a^ | 1980 – 2013 | NPR | 1378 | <15 | - | 2.4 | 3.3 | - | - | - |
|  |  |  |  | 13 697 | 15 – 29 | - | 14.3 | 23.4 |  |  |  |
| Denmark | Nørgård et al [7] (2014) | 1995 – 2012 | NPR | 940 | <15 | - | 2.8 (females)  3.3  (males) | 3.0 (females)  2.4  (males) | - | - | - |
|  |  |  |  |  |  |  |  |  |  |  |  |
|  |  |  |  | 7411 | 15 – 29 | - | 20.6 (females)  13.7  (males) | 32.0 (females)  26.9  (males) |  |  |  |
| Denmark (Eastern County) | Urne et al [8] (2002) | 1998 – 2000 | Health records | 98 | <15 | 4.3 | 2.3 | 1.8 | 15.8 | 6.7 | 8.3 |
| Denmark (Copenhagen) | Vind et al [9] (2006)^b,c^ | 2003 – 2005 | Health records | 562 | <18 | - | 4.4 | 5.0 | - | - | - |
|  |  |  |  |  | 16 – 25 | - | 19 | 19.5 |  |  |  |
| Finland | Jussila et al [10] (2012) ^b,c^ | 2000 – 2007 | NPR | 14 214 | 7 – 14 | - | 4.0 (females)  6.5 (males) | 9.0 (females)   9.5 (males) | - | - | - |
|  |  |  |  |  | 15 – 25 | - | 14.0 (females)  16.0 (males) | 32.5 (females)  35.0 (males) | - | - | - |
| Finland | Lehtinen et al [11] (2011) | 1987 – 2003 | Health records | 1880 | <18 | 15.0 | 5.0 | 9.0 | - | - | - |
| Finland (Helsinki and Tampere) | Turunen et al [12] (2006) | 1987 – 2003 | Health records | 604 | <18 | 7.0 | 2.6 | 3.2 | - | - | - |
| Finland | Virta et al [13] (2017) | 1987 – 2014 | NPR | 5415 | <20 | 23.0 | 8.0 | 15.0 | - | - | - |
| Iceland | Agnarsson et al [14] (2013)^b^ | 2001 – 2010 | Health records | 110 | <16 | 5.0 | 2.3 | 2.4 | - | - | - |
| Iceland | Björnsson et al [15] (2000) ^b,c^ | 1990 – 1994 | Health records | 287 | <19 | - | 9 | 10 | - | - | - |
|  |  |  |  |  | 20 – 29 | - | 10 | 28 |  |  |  |
| Norway (Oslo) | Perminow et al [16] (2006) | 1993 – 2004 | Health records | 48 | <16 | 5.7 | 2.8 | 2.8 | - | - | - |
| Norway (Southeast County) | Perminow et al [17] (2009) | 2005 – 2007 | Cohort study | 62 | <18 | 10.9 | 6.8 | 3.6 | - | - | - |
| Norway (Southeast County) | [Størdal](https://pubmed.ncbi.nlm.nih.gov/?size=100&term=St%C3%B8rdal+K&cauthor_id=15627770) et al [18]  (2004)^d^ | 1990 – 1993 | Cohort study | 33 | <16 | 4.7 | 2.7 | 2.0 | - | - | - |
| Sweden (Stockholm) | Askling et al [19]  (1999) | 1990 – 1998 | Health records | 91 | <17 | 6.9 | 3.8 | 2.1 | - | - | - |
| Sweden (Stockholm) | Hildebrand et al [20] (2003) | 1990 – 2001 | Health records | 152 | <15 | 7.4 | 4.9 | 2.2 | - | - | - |
| Sweden | Ludvigsson et al [21] (2017) | 1993 – 2010 | NPR | 1432 | <18 | - | - | - | 75 | 29 | 30 |
| Sweden (Stockholm) | Malmborg et al [22] (2013) | 2002 – 2007 | Health records | 133 | <16 | 12.8 | 9.2 | 2.8 | - | - | - |
| Sweden (Uppsala) | Rönnblom et al [23] (2010) ^b,c^ | 2005 – 2007 | Health records | 165 | 10 – 19 | - | - | 19.0 | - | - | - |
|  |  |  |  |  | 20 – 29 | - | - | 30.5 | - | - | - |
| Sweden (Uppsala) | Sjöberg et al [24] (2014)^c,e^ | 2005 – 2009 | Health records | 50 | <17 | - | 10.0 |  | - | - | - |
|  |  |  |  |  | 20-29 | - | 24.0 (females)  10.5 (males) |  |  |  |  |

^a^Different criteria for case definitions was presented, here we presented results for >2 records of IBD. ^b^Number of participants was only presented for the entire study population. ^c^Incidence and/or prevalence were calculated from graphs presented in the published papers

^d^Størdal et al 2004 [18] was a part of the Inflammatory Bowel Disease in South Eastern Norway (IBSEN) Study. Hence, the article by Bentsen et al 2002 [25] was not included as it was based on the same data (IBSEN I). ^e^Number of events were not presented for the age group of 20-29 years. Studies were ordered by country. Prevalence of IBD relates to all children ages 0-<15 years (Denmark) and 0-<18 years (Sweden). Source of diagnosis: Cohort study, e.g., prospective cohort studies, Health records, e.g., medical records; NPR National patient register, e.g., Swedish National Patient Register. *CD* Crohn's disease, *IBD* inflammatory bowel disease, *PYR* person-year, *UC* ulcerative colitis

**Table 2.** International Classification of Disease (ICD)-codes of inflammatory bowel disease

| Disease | ICD-10 (1997-) |
| --- | --- |
| **Inflammatory bowel disease** | K52.3, CD + UC or mixed diagnosis of CD, UC and IBD-U |
| **Crohn's disease** | K50 |
| **Ulcerative colitis** | K51 |
| **IBD-unclassified** | K52.3 |

**Table 3.** Disease phenotype^a^ of inflammatory bowel disease at time of diagnosis

| **Characteristics** | **Crohn's disease**  **N = 27** | **Ulcerative colitis N = 27** |
| --- | --- | --- |
| **Disease location, n (%)** |  |  |
| Ileal (L1) | 9 (33%) | - |
| Colonic (L2) | 10 (37%) | - |
| Ileocolonic (L3) | 7 (26%) | - |
| *Missing data* | 1 (4%) | - |
| **Disease behaviour, n (%)** |  |  |
| Non-stricturing, nonpenetrating (B1) | 23 (85%) | - |
| Stricturing (B2) | 1 (4%) | - |
| Penetrating (B3) | 2 (7%) | - |
| *Missing data* | 1 (4%) | - |
| **Disease extent, n (%)** |  |  |
| Proctitis (E1) | - | 6 (22%) |
| Left-sided (E2) | - | 5 (19%) |
| Extensive colitis (E3) | - | 14 (52%) |
| *Missing data* | - | 2 (7%) |

^a^According to the Montreal classification. Data restricted to 57 patients who had a validated (true) inflammatory bowel disease diagnosis on medical record review. Three patients with undefined inflammatory bowel disease (IBD) were not specifically reported in the table: one with undefined IBD had left-sided colitis (E2) and two had extensive colitis (E3).

**Table 4.** Disease phenotype^a^ of inflammatory bowel disease for patients aged ≤16 years at the time of diagnosis

| **Characteristics** | **Crohn's disease**  **N = 18** | **Ulcerative colitis N = 19** |
| --- | --- | --- |
| **Disease location, n (%)** |  |  |
| Ileal (L1) | 7 (39%) | - |
| Colonic (L2) | 6 (33%) | - |
| Ileocolonic (L3) | 5 (28%) | - |
| *Missing data* | 0 (0%) | - |
| **Disease behaviour, n (%)** |  |  |
| Non-stricturing, nonpenetrating (B1) | 15 (83%) | - |
| Stricturing (B2) | 1 (6%) | - |
| Penetrating (B3) | 2 (11%) | - |
| *Missing data* | 0 (0%) | - |
| **Disease extent, n (%)** |  |  |
| Proctitis (E1) | - | 4 (21%) |
| Left-sided (E2) | - | 5 (26%) |
| Extensive colitis (E3) | - | 9 (47%) |
| *Missing data* | - | 1 (5%) |

^a^According to the Montreal classification. Data restricted to 57 patients who had a validated (true) inflammatory bowel disease diagnosis on medical record review. Three patients with undefined inflammatory bowel disease were not specifically reported in the table: one with undefined IBD had left-sided colitis (E2) and two had extensive colitis (E3).

**Table 5.** Disease phenotype^a^ of inflammatory bowel disease for patients aged 17 years or older at the time of diagnosis

| **Characteristics** | **Crohn's disease**  **N = 9** | **Ulcerative colitis N = 8** |
| --- | --- | --- |
| **Disease location, n (%)** |  |  |
| Ileal (L1) | 2 (22%) | - |
| Colonic (L2) | 4 (44%) | - |
| Ileocolonic (L3) | 2 (22%) | - |
| *Missing data* | 1 (11%) | - |
| **Disease behaviour, n (%)** |  |  |
| Non-stricturing, nonpenetrating (B1) | 8 (89%) | - |
| Stricturing (B2) | 0 (0%) | - |
| Penetrating (B3) | 0 (0%) | - |
| *Missing data* | 1 (11%) | - |
| **Disease extent, n (%)** |  |  |
| Proctitis (E1) | - | 2 (25%) |
| Left-sided (E2) | - | 0 (0%) |
| Extensive colitis (E3) | - | 5 (63%) |
| *Missing data* | - | 1 (13%) |

^a^According to the Montreal classification. Data restricted to 57 patients who had a validated (true) inflammatory bowel disease diagnosis on medical record review.

**Table 6.** Medical therapy^a^ within the first year of diagnosis for inflammatory bowel disease patients aged ≤16 years at the time of diagnosis

| **Therapy** | **Crohn's disease**  **N = 18** | **Ulcerative colitis N = 19** | **IBD-unclassified**  **N = 3** |
| --- | --- | --- | --- |
| **Oral corticosteroids** |  |  |  |
| Yes | 15 (83%) | 12 (63%) | 3 (100%) |
| No | 1 (6%) | 4 (21%) | 0 (0%) |
| *Missing data* | 2 (11%) | 3 (16%) | 0 (0%) |
| **Oral 5-ASA** |  |  |  |
| Yes | 13 (72%) | 11 (58%) | 2 (67%) |
| No | 3 (17%) | 5 (26%) | 1 (33%) |
| *Missing data* | 2 (11%) | 3 (16%) | 0 (0) |
| **Azathioprine** |  |  |  |
| Yes | 14 (78%) | 7 (37%) | 1 (67%) |
| No | 2 (11%) | 9 (47%) | 2 (33%) |
| *Missing data* | 2 (11%) | 3 (16%) | 0 |
| **Biologics** |  |  |  |
| Yes | 5 (28%) | 1 (6%) | 0 (0%) |
| No | 11 (61%) | 15 (79%) | 3 (100%) |
| *Missing data* | 2 (11%) | 3 (16%) | 0 (0%) |

Three patients ≤16 years old with undefined inflammatory bowel disease (IBD) were not specifically reported in the table: Three had oral corticosteroids, two with undefined IBD had 5-aminosalicylic acid (5-ASA), one had azathioprine and none of those three with undefined IBD had biologics.

**Table 7.** Medical therapy^a^ within the first year of diagnosis for inflammatory bowel disease patients aged 17 years or older at the time of diagnosis

| **Therapy** | **Crohn's disease**  **N = 9** | **Ulcerative colitis N = 8** |
| --- | --- | --- |
| **Oral corticosteroids** |  |  |
| Yes | 9 (100%) | 12 (38%) |
| No | 0 (0%) | 4 (50%) |
| *Missing data* | 0 (0%) | 3 (13%) |
| **Oral 5-ASA** |  |  |
| Yes | 5 (56%) | 6 (75%) |
| No | 4 (44%) | 1 (13%) |
| *Missing data* | 0 (0%) | 1 (13%) |
| **Azathioprine** |  |  |
| Yes | 7 (78%) | 2 (25%) |
| No | 2 (11%) | 5 (63%) |
| *Missing data* | 0 (11%) | 1 (13%) |
| **Biologics** |  |  |
| Yes | 4 (44%) | 0 (0%) |
| No | 5 (66%) | 7 (88%) |
| *Missing data* | 0 (0%) | 1 (13%) |

**
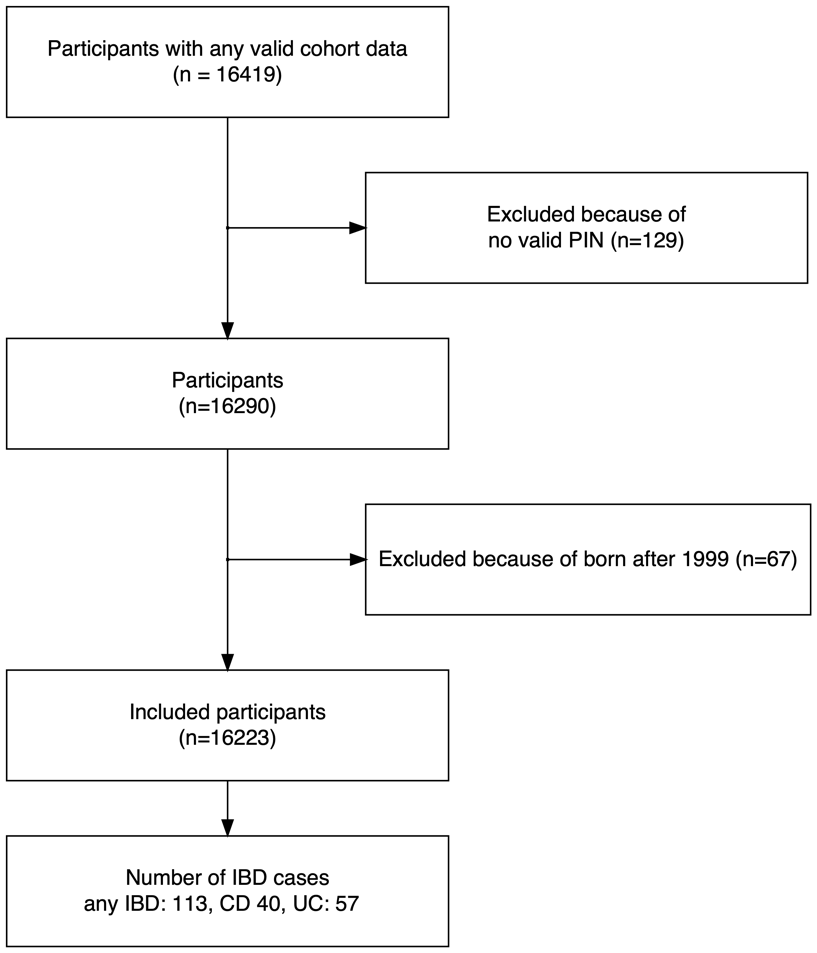
**

**Figure 1.** Formation of ABIS cohort and participants with a register-based definition of inflammatory bowel disease (IBD, ≥2 diagnostic listings) using data from the Sweden National Patient Register until December 31, 2020. Children without valid personal identification number (PIN) were excluded as PIN is required for register linkages. We also excluded 67 children born after 1999 as they were not considered part of the source population of ABIS (births between October 1, 1997, and October 1, 1999). Among the 113 participants with any IBD, 16 undefined IBD. *CD* Crohn's disease, *UC* ulcerative colitis

**REFERENCES**

1. Jacobsen BA, Fallingborg J, Rasmussen HH, Nielsen KR, Drewes AM, Puho E, Nielsen GL, Sørensen HT: **Increase in incidence and prevalence of inflammatory bowel disease in northern Denmark: a population-based study, 1978-2002**. *Eur J Gastroenterol Hepatol* 2006, **18**(6):601-606.

2. Jakobsen C, Wewer V, Urne F, Andersen J, Faerk J, Kramer I, Stagegaard B, Pilgaard B, Weile B, Paerregaard A: **Incidence of ulcerative colitis and Crohn's disease in Danish children: Still rising or levelling out?** *J Crohns Colitis* 2008, **2**(2):152-157.

3. Jakobsen C, Paerregaard A, Munkholm P, Wewer V: **Paediatric inflammatory bowel disease during a 44-year period in Copenhagen County: occurrence, course and prognosis--a population-based study from the Danish Crohn Colitis Database**. *Eur J Gastroenterol Hepatol* 2009, **21**(11):1291-1301.

4. Larsen MD, Baldal ME, Nielsen RG, Nielsen J, Lund K, Nørgård BM: **The incidence of Crohn's disease and ulcerative colitis since 1995 in Danish children and adolescents <17 years - based on nationwide registry data**. *Scand J Gastroenterol* 2016, **51**(9):1100-1105.

5. Larsen L, Karachalia Sandri A, Fallingborg J, Jacobsen BA, Jacobsen HA, Bøgsted M, Drewes AM, Jess T: **Has the Incidence of Inflammatory Bowel Disease Peaked? Evidence From the Population-Based NorDIBD Cohort 1978-2020**. *Am J Gastroenterol* 2023, **118**(3):501-510.

6. Lophaven SN, Lynge E, Burisch J: **The incidence of inflammatory bowel disease in Denmark 1980-2013: a nationwide cohort study**. *Aliment Pharmacol Ther* 2017, **45**(7):961-972.

7. Nørgård BM, Nielsen J, Fonager K, Kjeldsen J, Jacobsen BA, Qvist N: **The incidence of ulcerative colitis (1995-2011) and Crohn's disease (1995-2012) - based on nationwide Danish registry data**. *J Crohns Colitis* 2014, **8**(10):1274-1280.

8. Urne FU, Paerregaard A: **[Chronic inflammatory bowel disease in children. An epidemiological study from eastern Denmark 1998-2000]**. *Ugeskr Laeger* 2002, **164**(49):5810-5814.

9. Vind I, Riis L, Jess T, Knudsen E, Pedersen N, Elkjaer M, Bak Andersen I, Wewer V, Nørregaard P, Moesgaard F *et al*: **Increasing incidences of inflammatory bowel disease and decreasing surgery rates in Copenhagen City and County, 2003-2005: a population-based study from the Danish Crohn colitis database**. *Am J Gastroenterol* 2006, **101**(6):1274-1282.

10. Jussila A, Virta LJ, Kautiainen H, Rekiaro M, Nieminen U, Färkkilä MA: **Increasing incidence of inflammatory bowel diseases between 2000 and 2007: a nationwide register study in Finland**. *Inflamm Bowel Dis* 2012, **18**(3):555-561.

11. Lehtinen P, Ashorn M, Iltanen S, Jauhola R, Jauhonen P, Kolho KL, Auvinen A: **Incidence trends of pediatric inflammatory bowel disease in Finland, 1987-2003, a nationwide study**. *Inflamm Bowel Dis* 2011, **17**(8):1778-1783.

12. Turunen P, Kolho KL, Auvinen A, Iltanen S, Huhtala H, Ashorn M: **Incidence of inflammatory bowel disease in Finnish children, 1987-2003**. *Inflamm Bowel Dis* 2006, **12**(8):677-683.

13. Virta LJ, Saarinen MM, Kolho KL: **Inflammatory Bowel Disease Incidence is on the Continuous Rise Among All Paediatric Patients Except for the Very Young: A Nationwide Registry-based Study on 28-Year Follow-up**. *J Crohns Colitis* 2017, **11**(2):150-156.

14. Agnarsson U, Björnsson S, Jóhansson JH, Sigurdsson L: **Inflammatory bowel disease in Icelandic children 1951-2010. Population-based study involving one nation over six decades**. *Scand J Gastroenterol* 2013, **48**(12):1399-1404.

15. Björnsson S, Jóhannsson JH: **Inflammatory bowel disease in Iceland, 1990-1994: a prospective, nationwide, epidemiological study**. *Eur J Gastroenterol Hepatol* 2000, **12**(1):31-38.

16. Perminow G, Frigessi A, Rydning A, Nakstad B, Vatn MH: **Incidence and clinical presentation of IBD in children: comparison between prospective and retrospective data in a selected Norwegian population**. *Scand J Gastroenterol* 2006, **41**(12):1433-1439.

17. Perminow G, Brackmann S, Lyckander LG, Franke A, Borthne A, Rydning A, Aamodt G, Schreiber S, Vatn MH: **A characterization in childhood inflammatory bowel disease, a new population-based inception cohort from South-Eastern Norway, 2005-07, showing increased incidence in Crohn's disease**. *Scand J Gastroenterol* 2009, **44**(4):446-456.

18. Størdal K, Jahnsen J, Bentsen BS, Moum B: **Pediatric inflammatory bowel disease in southeastern Norway: a five-year follow-up study**. *Digestion* 2004, **70**(4):226-230.

19. Askling J, Grahnquist L, Ekbom A, Finkel Y: **Incidence of paediatric Crohn's disease in Stockholm, Sweden**. *Lancet* 1999, **354**(9185):1179.

20. Hildebrand H, Finkel Y, Grahnquist L, Lindholm J, Ekbom A, Askling J: **Changing pattern of paediatric inflammatory bowel disease in northern Stockholm 1990-2001**. *Gut* 2003, **52**(10):1432-1434.

21. Ludvigsson JF, Büsch K, Olén O, Askling J, Smedby KE, Ekbom A, Lindberg E, Neovius M: **Prevalence of paediatric inflammatory bowel disease in Sweden: a nationwide population-based register study**. *BMC Gastroenterol* 2017, **17**(1):23.

22. Malmborg P, Grahnquist L, Lindholm J, Montgomery S, Hildebrand H: **Increasing incidence of paediatric inflammatory bowel disease in northern Stockholm County, 2002-2007**. *J Pediatr Gastroenterol Nutr* 2013, **57**(1):29-34.

23. Rönnblom A, Samuelsson SM, Ekbom A: **Ulcerative colitis in the county of Uppsala 1945-2007: incidence and clinical characteristics**. *J Crohns Colitis* 2010, **4**(5):532-536.

24. Sjöberg D, Holmström T, Larsson M, Nielsen AL, Holmquist L, Ekbom A, Rönnblom A: **Incidence and clinical course of Crohn's disease during the first year - results from the IBD Cohort of the Uppsala Region (ICURE) of Sweden 2005-2009**. *J Crohns Colitis* 2014, **8**(3):215-222.

25. Bentsen BS, Moum B, Ekbom A: **Incidence of inflammatory bowel disease in children in southeastern Norway: a prospective population-based study 1990-94**. *Scand J Gastroenterol* 2002, **37**(5):540-545.
